# The osteogenic and mineralogenic potential of the microalgae *Skeletonema costatum* and *Tetraselmis striata* CTP4 in fish models

**DOI:** 10.1007/s00018-023-04953-y

**Published:** 2023-09-30

**Authors:** Alessio Carletti, Joana T. Rosa, Katia Pes, Inês Borges, Tamára Santos, Luísa Barreira, João Varela, Hugo Pereira, M. Leonor Cancela, Paulo J. Gavaia, Vincent Laizé

**Affiliations:** 1 Centre of Marine Sciences (CCMAR), University of Algarve, Faro, Portugal; 2 Faculty of Medicine and Biomedical Sciences (FMCB), University of Algarve, Faro, Portugal; 3 Present Address: Collaborative Laboratory for Sustainable and Smart Aquaculture (S2AQUAcoLAB), Olhão, Portugal; 4 Associação Oceano Verde (GreenCoLab), Faro, Portugal; 5 Algarve Biomedical Center (ABC), University of Algarve, Faro, Portugal

**Keywords:** Marine microalgae, Osteoanabolic activity, Mineralogenic cell line, Skeletal deformities, Zebrafish, Gilthead seabream

## Abstract

Skeletal disorders are problematic aspects for the aquaculture industry as skeletal deformities, which affect most species of farmed fish, increase production costs and affect fish welfare. Following recent findings that show the presence of osteoactive compounds in marine organisms, we evaluated the osteogenic and mineralogenic potential of commercially available microalgae strains *Skeletonema costatum* and *Tetraselmis striata* CTP4 in several fish systems. Ethanolic extracts increased extracellular matrix mineralization in gilthead seabream (*Sparus aurata*) bone-derived cell cultures and promoted osteoblastic differentiation in zebrafish (*Danio rerio*) larvae. Long-term dietary exposure to both extracts increased bone mineralization in zebrafish and upregulated the expression of genes involved in bone formation (*sp7*, *col1a1a*, *oc1*, and *oc2*), bone remodeling (*acp5a*), and antioxidant defenses (*cat*, *sod1*). Extracts also improved the skeletal status of zebrafish juveniles by reducing the incidence of skeletal anomalies. Our results indicate that both strains of microalgae contain osteogenic and mineralogenic compounds, and that ethanolic extracts have the potential for an application in the aquaculture sector as dietary supplements to support fish bone health. Future studies should also identify osteoactive compounds and establish whether they can be used in human health to broaden the therapeutic options for bone erosive disorders such as osteoporosis.

**Supplementary Information:**

The online version contains supplementary material available at 10.1007/s00018-023-04953-y.

## Introduction

Osteogenic compounds that can stimulate bone formation and mineralization have received increasing attention by researchers in the field of aquaculture due to the existence of skeletal disorders that significantly impact the value and welfare of farmed fish. High incidences of skeletal anomalies are observed in virtually all species of reared fish. These anomalies alter fish swimming behavior and feeding abilities, thus diminish welfare and survival, but also negatively impact on customer perception and sales, thus limit profits for the industry [1, 2]. The supplementation of fish diets with ingredients that support a healthy skeletal development is seen as a suitable strategy to reduce the incidence of skeletal anomalies in aquaculture species [2]. Natural compounds with the capacity to induce bone formation and mineralization (i.e., osteoanabolic compounds) have been uncovered in a variety of organisms, including marine organisms [3–7]; these compounds have the potential to be the basis of new dietary supplements to improve the skeletal health of farmed fish [8].

In this scenario, marine microalgae have gained momentum in applications related to the blue economy and sustainable growth [9], ranging from clean energy production [10], wastewater treatment [11], animal feeds [12, 13] to human nutrition and medicine [14, 15]. They are optimal sources of essential nutrients [16], and being able to produce a plethora of bioactive molecules [17], they are expected to fuel pharmaceutical development in the upcoming years [18]. The increasing interest of academia and industries for microalgae has stimulated the development of methods for the large-scale production of biomass and genetic engineering tools have recently been successfully applied to microalgae, allowing for the development of transgenic lines optimized for the synthesis of specific compounds, shaping the idea of microalgae as full-fledge bioreactors for the production of biomolecules [19–21].

Despite a growing interest for microalgae-derived bioactives, very few studies have explored microalgae as a source of osteogenic and mineralogenic compounds, possibly because of the perceived lack of suitable systems to assess these bioactivities. In this regard, small teleosts such as the zebrafish (*Danio rerio*) presents several technical advantages over classical mammalian models, including lower maintenance costs, small size, short life cycle, high fecundity, easier genetic manipulation, and translucent embryonic stages [22–27]. For the same advantages provided to biomedical research and in virtue of its evolutionary resemblance with teleosts of commercial interest, zebrafish is also becoming a popular model for aquaculture research, being successfully implemented for research in fish nutrition and immunology [28, 29]. Several fish-based in vitro and in vivo systems have been recently described [30–35] and successfully used to assess the presence of osteoactive compounds found in marine invertebrates, seaweeds, and halophyte plants [3–7]. In the present work, extracts from two commercially available species of marine microalgae—*Skeletonema costatum* and *Tetraselmis striata* CTP4—were prepared through ethanolic maceration and evaluated for the presence of osteogenic and mineralogenic compounds using a diverse set of screening systems.

## Materials and methods

### Preparation of ethanolic extracts

**Fig. 1:**
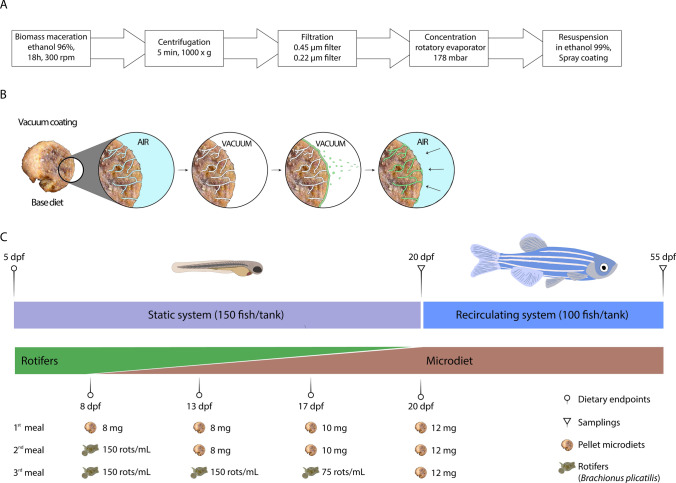
**A** Schematic representation of the production of microalgal extracts. **B** Vacuum coating of commercial diets with algal extracts. **C** Time course and feeding table of the nutritional trial. *rots* rotifers

Freeze-dried biomass of *Skeletonema costatum* (Necton S.A., Olhão, Portugal) and *Tetraselmis striata* CTP4 (ALLMICROALGAE—Natural Products S.A., Pataias, Portugal) was macerated with 96% ethanol (Merck, Darmstadt, Germany) at a ratio of 1 g of biomass for 40 mL of solvent, under gentle agitation at 24 °C for 18 h. Macerates were centrifuged for 5 min at 1000×g and supernatants were collected. Pellets were washed twice with 96% ethanol and supernatants were pooled and then vacuum filtered sequentially through 0.45 µm and 0.22 µm nylon membranes (Labbox Labware S.L. Barcelona, Spain). Filtrates were kept at – 20 °C until concentrated using a rotatory evaporator RV 10 digital (IKA-Werke GmbH & Co, Staufen im Breisgau, Germany) at 40 °C. Extracts were evaporated until a dense paste was formed. Extraction yields were determined from 2 mL of each concentrated solution placed under a gentle flow of 99.8% nitrogen until complete evaporation. The yield of the extraction process was 37.9% for *S. costatum* and 19.6% for *T. striata* CTP4 (Fig. 1A).

### Cell culture and extracellular matrix mineralization

A previously established gilthead seabream (*Sparus aurata*) line of osteochondroprogenitor cells (VSa13; Cellosaurus accession number CVCL_S952) [30] was maintained in Dulbecco’s modified Eagle medium (DMEM) supplemented with 10% fetal bovine serum, 1% penicillin–streptomycin, 1% L-glutamine, and 0.2% fungizone (all from GIBCO, ThermoFisher Scientific, Waltham, USA) at 33 °C in a 10% CO_2_-humidified atmosphere [30, 31]. Pre-confluent cell cultures were sub-cultured 1:4 twice a week using trypsin–EDTA solution (0.2% trypsin from GIBCO, 1.1 mM EDTA, pH 7.4). Mineralization assays were conducted in 48-well plates seeded at a density of 1.25 × 10^4^ cells per well (1.56 × 10^4^ cells/cm^2^) and incubated until culture reached confluency. Extracellular matrix (ECM) mineralization was induced by supplementing culture medium with L-ascorbic acid (50 μg/mL), β-glycerophosphate (10 mM) and calcium chloride (4 mM). Mineralizing cell cultures were treated with ethanolic extracts from *Skeletonema costatum* or *Tetraselmis striata* CTP4 (thereafter referred to as SKLT and CTP4) at different concentrations, or with their vehicle (0.1% ethanol), thereafter designed as the control group (CTRL). Cells cultured in non-supplemented medium (i.e., without mineralogenic cocktail and extracts) were also used as a negative control (Min^−^). After 17 days of culture, mineral deposition was assessed through alizarin red S (AR-S, Sigma-Aldrich, St. Louis, USA) staining and quantified by spectrophotometry as previously described [30].

### Zebrafish maintenance

Zebrafish wild-type (AB) and transgenic lines *Tg(Hsa.RUNX2-Mmu.Fos:EGFP)*^*zf259*^, *Tg(Ola.Sp7:mCherry-Eco.NfsB)*^*pd46*^ and *Tg(Ola.osteocalcin:EGFP)*^*hu4008*^, hereafter referred to as *Tg(runx2:EGFP)*, *Tg(sp7:mCherry)* and *Tg(oc:EGFP)* [36, 37], were maintained in a ZebTEC water recirculating system (Tecniplast, Buguggiate, Italy) at the aquatic animal facilities of the Centre of Marine Sciences (CCMAR, Faro, Portugal). Eggs were obtained following an in-house breeding program using sexually mature adult zebrafish. Viable fertilized eggs were transferred into plastic 1 L tanks and maintained for 3 days in reverse osmosis-treated water supplemented with salts (Instant Ocean, Blacksburg, USA), sodium bicarbonate (Fisher Chemicals, Hampton, USA), and methylene blue (0.0002% w/v, Sigma-Aldrich). Water parameters were as following: temperature 28 ± 0.1 °C, pH 7.5 ± 0.1, conductivity 700 ± 50 μS/cm, ammonia and nitrites lower than 0.1 mg/L, and nitrates at 5 mg/L. Photoperiod was set to 14:10 h light–dark.

### Waterborne exposure of zebrafish larvae to microalgae extracts

At 3 days post-fertilization (dpf), hatched larvae from AB and transgenic lines were transferred to 6-well plates at a density of 15 larvae/well, and exposed to ethanolic extracts, 0.1% ethanol (vehicle and negative control) or 10 pg/mL of calcitriol (1α,25-dihydroxy vitamin D_3_, Sigma-Aldrich; positive control) [33]. Treatment (10 mL/well) was renewed—70% of the total volume, i.e., 7 mL—daily until the end of the experiment. Mortality was monitored at the time of treatment renewal in order to establish the highest non-toxic concentration for each extract. At 6 dpf, larvae were killed with a lethal dose of MS-222 (0.6 mM, pH 7.0, Sigma-Aldrich), then stained for 20 min with 0.03% alizarin red S or 0.5% calcein prepared in Milli-Q water (pH 7.4; Millipore, Burlington, USA) and washed twice with Milli-Q water for 5 min. Larvae were imaged immediately upon staining. For gene expression analysis, 3-dpf larvae (AB line; 10 larvae per replicate and 3 replicates) were placed in a 100 × 15 mm Petri dish (Sarstedt) with 35 mL of treatment (prepared as described above). Larvae were exposed for 3 days to the highest non-toxic concentration of each extract, or the vehicle. At 6 dpf, fish from each Petri dish were pooled (10 fish per pool; 3 pools per condition) and processed for RNA extraction (see below).

### Imaging and morphometric analysis

Euthanized larvae were placed in a lateral position on top of a 2.5% agarose gel plate and imaged using an MZ10F fluorescence stereomicroscope (Leica, Wetzlar, Germany) equipped with a DFC7000T color camera (Leica). A green fluorescence filter (*λ*ex = 546/10 nm) and a barrier filter (*λ*em = 590 nm) were used to image AR-S stained wild-type larvae and *Tg(sp7:mCherry)* transgenic larvae, while a blue fluorescence filter (λex = 470/40 nm) and a barrier filter (*λ*em = 515 nm) were used to image calcein stained wild-type larvae, and *Tg(runx2:EGFP)* and *Tg(oc:EGFP)* transgenic larvae. Images were acquired using the following parameters: exposure time of 300 ms (mCherry and AR-S) or 1 s (calcein and EGFP); gamma 1.00; resolution 1920 × 1440 pixels; binning 1 × 1. Images were analyzed using ImageJ (version 1.52p). For morphometric analysis, images were processed using ZFBONE macro-toolset for Fiji [34]. The area of the operculum (OpA), the area of the head (HA), the splanchnocranial area (SA), and the areas positive for reporter signals within the HA (*sp7*^+^, *oc*^+^) and within the SA (*runx2*^+^) were determined using an Intuos M drawing tablet (Wacom, Kazo, Japan). To correct for inter-specimen size variations OpA, *sp7*^+^ and *oc*^+^ were normalized using HA, and *runx2*^+^ was normalized using SA.

### Preparation of feeds supplemented with ethanolic extracts

Ethanolic extracts SKLT and CTP4 were vacuum coated onto a zebrafish base diet (Sparos Lda, Olhão, Portugal) at 0.5% and 2.5% each. The five diets—CTRL (no extract), SKLT 0.5%, SKLT 2.5%, CTP4 0.5%, and CTP4 2.5% (Fig. 1B) were analyzed for phosphorus (P), calcium (Ca), potassium (K), and magnesium (Mg) contents. For this, diets were completely dried at 120 °C for 24 h in a VENTI-Line drying oven (VWR International, Radnor, USA), weighted and solubilized in nitric acid (Sigma-Aldrich). Mineral content was determined by microwave plasma atomic emission spectroscopy (4200 MP-AES, Agilent Technologies, Santa Clara, USA) using P, Ca, K, and Mg standards.

### Dietary exposure to ethanolic extracts

At 3 dpf, zebrafish AB larvae were randomly distributed into 2.5 L tanks filled with water from the recirculating system filtered using 0.22 µm nylon membranes (Labbox) and maintained in static conditions at a density of 60 larvae/L. Environmental parameters were set as described above. Experimental procedures were conducted in triplicates for all conditions and the feeding regimen was adjusted with the growth of the fish (Fig. 1C). From 5 to 7 dpf, larvae were fed exclusively with live rotifers (*Brachionus plicatilis*). From 8 to 19 dpf, larvae were maintained in a regimen of co-feeding with rotifers and the experimental diets, gradually decreasing the number of rotifers and increasing the amount of dry food. From 20 to 55 dpf, fish were fed three times per day solely with the experimental diets. Food size was < 100 µm until 16 dpf and 100–200 µm until 55 dpf. At 20 dpf, 50 fish/tank were sampled to determine the total length (TL), while the remaining fish were transferred to 2.8 L glass tanks and maintained in recirculating water conditions until 55 dpf at a density of 36 larvae/L. At the end of the trial, juveniles were given a lethal anesthesia with MS-222 then randomly pooled for the assessment of dry weight (4 pools/replicate, 3 fish/pool), content of Ca and P (2 pools/replicate, 3 fish/pool), and gene expression by qPCR (2 pools/replicate, 3 fish/pool), as described below. The remaining fish (≥ 30 fish/replicate) were placed in a lateral position over a 2.5% agarose gel and photographed under a Leica MZ10F stereomicroscope equipped with a Leica DFC7000T color camera. Total length was assessed from bright-field images using ImageJ, then fish were fixed for 16 h in 4% paraformaldehyde prepared in phosphate-buffered saline (PBS, pH 7.4), then washed with PBS for 5 min and dehydrated in an increasing ethanol series. Subsequently, specimens were stained for 20 min with 0.05% AR-S in 1% KOH, then washed in 1% KOH for 48 h and preserved in glycerol. Incidence of skeletal anomalies and rate of skeleton mineralization were assessed in stained fish. To estimate the mineralization status of the skeleton, a color code was attributed to each skeletal element according to a mineralization index, where 0 is for elements with no mineralization, 1 for elements with a low level of mineralization, 2 for elements with intermediate levels of mineralization, and 3 for elements fully mineralized.

### RNA extraction and qPCR analysis

Total RNA was extracted from pools of larvae at 6 dpf (*n* = 3, 10 larvae/pool) or juvenile fish at 55 dpf (*n* = 6, 3 fish/pool) using NZYol (NZYTech, Lisbon, Portugal) and quantified using a NanoDrop OneC spectrophotometer (ThermoFisher Scientific). RNA integrity was confirmed using an Experion Automated Electrophoresis system (Bio-Rad, Hercules, USA). Total RNA (1 μg) was reverse-transcribed for 1 h at 37 °C using M-MLV reverse transcriptase, oligo-d(T) primer and RNAseOUT (all from ThermoFisher Scientific). Quantitative real-time PCR (qPCR) reactions were performed using qPCR NZYSpeedy Mastermix (2x) ROX Plus (NZYTech), 10 μM of gene-specific primers (Supplementary Table I) and 1:10 dilution of reverse-transcribed RNA, in a CFX Connect Real-Time PCR detection system (Bio-Rad). PCR amplification was as follows: an initial denaturation step of 2 min at 95 °C and 40 cycles of amplification (10 s at 95 °C and 20 s at 65 °C). Efficiency of amplification was above 95% for all primer sets. Primer specificity was confirmed through sequence analysis and qPCR specificity was assessed by melting curve analysis at the end of each PCR run. Levels of gene expression were calculated using the ∆∆Ct method [38] and normalized using the average value of three housekeeping genes, *ef1a*, *actb1* and *rps18*.

### Statistical analysis

For all the experiments, normality was tested with a D’Agostino–Pearson omnibus normality test or with an Anderson–Darling test (*p* < 0.05). Homoscedasticity was tested through the Brown–Forsythe test (*p* < 0.05). If data distribution was normal and homogeneous in all the experimental groups, differences were tested with an unpaired *t* test (gene expression, *p* < 0.05) or a one-way ANOVA followed by Dunnett’s multiple comparison test (all other parameters, *p* < 0.05). If data distribution was not normal or not homogeneous, differences were tested with a Mann–Whitney test (gene expression, *p* < 0.05) or a non-parametric test followed by Dunn’s multiple comparison test (all other parameters, *p* < 0.05). Statistical analyses were performed using Prism version 8.00 (GraphPad Software Inc., La Jolla, USA).

## Results

### Microalgae extracts induce matrix mineralization in fish bone cell lines

**Fig. 2:**
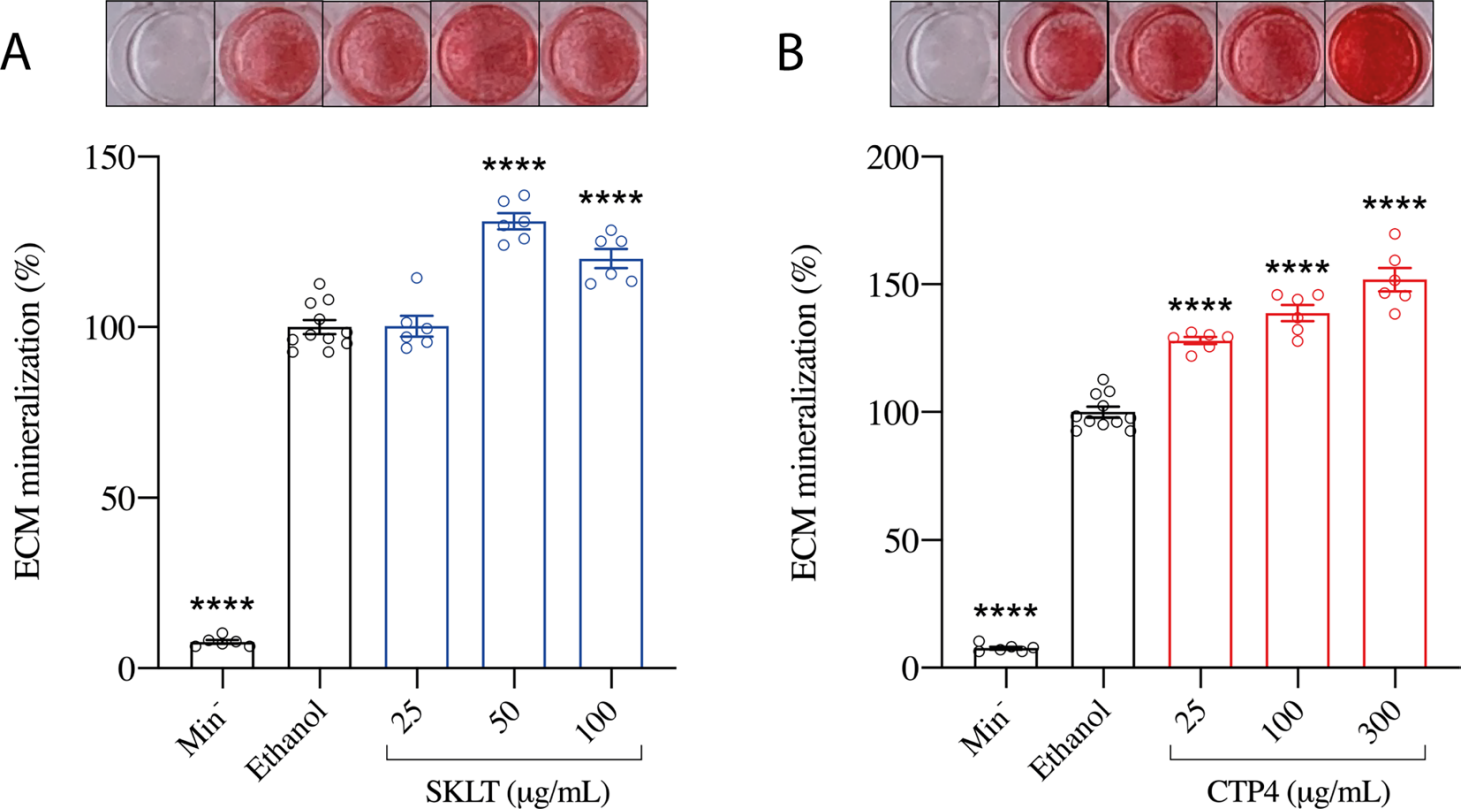
Mineralization of the extracellular matrix (ECM) of VSa13 cells exposed to ethanolic extracts prepared from **A** *Skeletonema costatum* (SKLT), and **B** *Tetraselmis striata* CTP4 (CTP4). Values are presented as mean ± standard deviation and as a percentage over the control group (Ethanol, *n* = 6). Picture panels above each graph display images of alizarin-red-stained cell cultures. Normality was tested through Anderson–Darling test (*p* < 0.05). Asterisks indicate values significantly different according to one-way ANOVA followed by post hoc Dunnett’s or Kruskal–Wallis test. Each experimental group was tested against the control group (Ethanol). *p* < 0.0001 (****). *Min*^*−*^ No mineralogenic cocktail

As a first approach to evaluate the osteogenic potential of the ethanolic extracts prepared from *Skeletonema costatum* (SKLT) and *Tetraselmis striata* CTP4 (CTP4), mineralogenic cells VSa13 were exposed for 17 days to 3 concentrations of the two extracts. While SKLT increased ECM mineralization at the two highest concentrations (50 and 100 µg/mL; Fig. 2A), CTP4 showed a strong mineralogenic activity at all the concentrations tested (25, 100, and 300 µg/mL; Fig. 2B) and in a dose-dependent manner, indicating the presence of mineralogenic compounds in both extracts.

### Microalgae extracts promote bone formation by stimulating osteoblastic differentiation in vivo

**Fig. 3:**
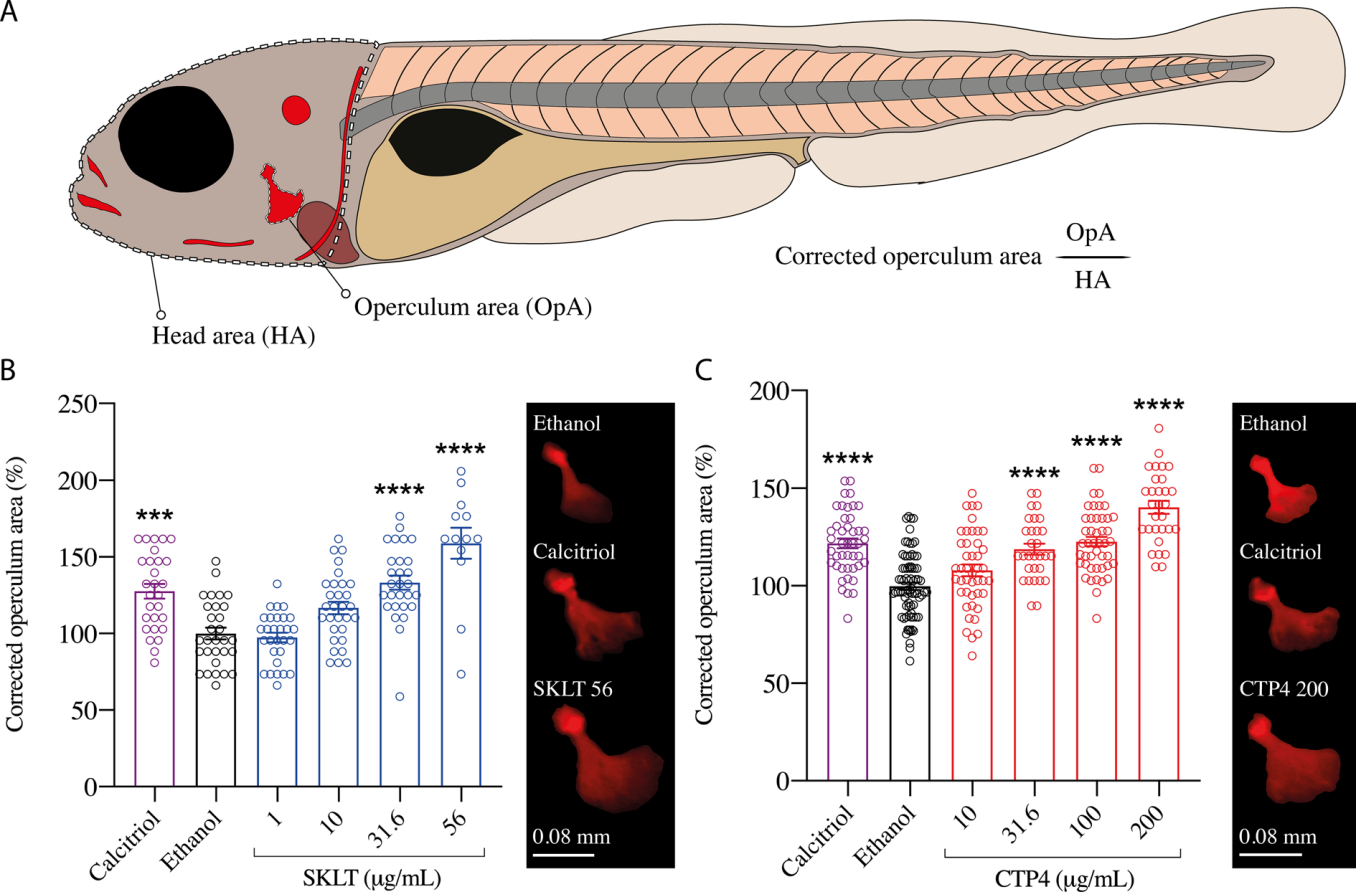
Mineralogenic effect on the operculum of 6-dpf zebrafish larvae exposed to *Skeletonema costatum* (SKLT) and *Tetraselmis striata* CTP4 (CTP4) extracts. **A** Composite image of a 6 dpf zebrafish larva, where operculum area (OpA) and head area (HA) are highlighted with a continuous line and a dashed line, respectively, and where AR-S stained mineralized structures detected by fluorescence microscopy appear in red. **B**, **C** Effects of SKLT (**B**) and CTP4 (**C**) on the operculum area. Values are presented as mean ± standard error and as a percentage over the negative control (Ethanol) (*n* > 15). The picture panels on the right side of each graph present images of opercular bones from negative and positive (calcitriol) control fish and fish exposed to the higher concentration of each extract. Normality was tested though Anderson–Darling test (*p* < 0.05). Asterisks indicate values significantly different according to one-way ANOVA followed by post hoc Dunnett’s or Kruskal–Wallis test. Each experimental group was tested against the negative control group (Ethanol). *p* < 0.0002 (***) and *p* < 0.0001 (****)

**Fig. 4:**
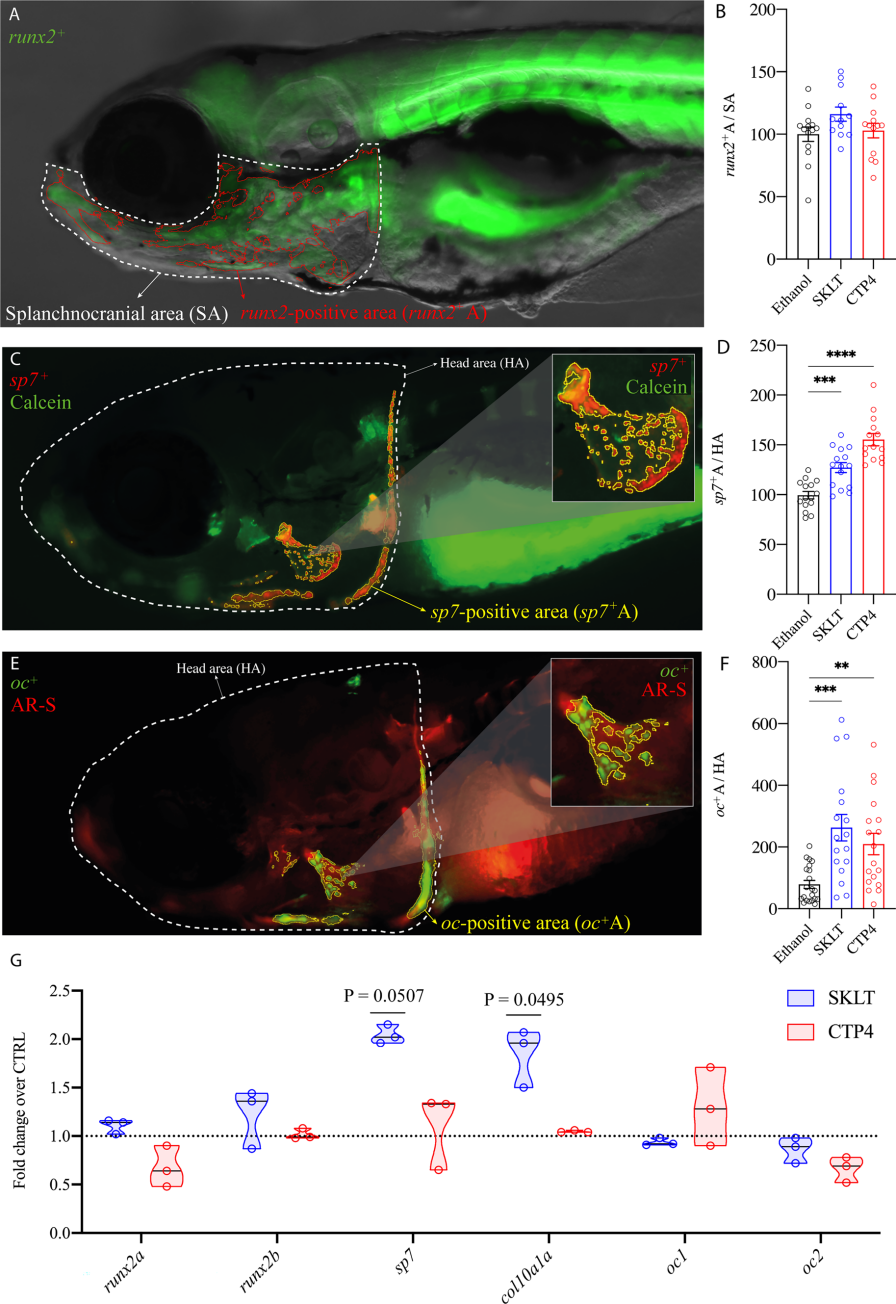
Expression of osteoblast differentiation markers in 6-dpf zebrafish larvae exposed to ethanolic extracts of *Skeletonema costatum* (SKLT) and *Tetraselmis striata* CTP4 (CTP4). Representative images of **A** *runx2*-positive osteochondroprogenitor cells, **C** *sp7*-positive immature osteoblasts, and **E** *oc1*-positive mature osteoblasts, and quantification of fluorescence signal area for *runx2* (**B**), *sp7* (**D**), and *oc1* (**E**) positive cells. Normality was tested though Anderson–Darling test (*p* < 0.05). For fluorescence signals, differences were tested through one-way ANOVA or Kruskal–Wallis test. Asterisks indicate *p* < 0.0021 (**), *p* < 0.0002 (***) and *p* < 0.0001 (****). For gene expression, differences were tested through a non-parametric Kruskal–Wallis test, followed by a Dunn’s multiple comparison test (significant P values are reported above each treatment)

The osteogenic potential of both extracts was assessed in zebrafish via waterborne exposure of 3-dpf larvae to different concentrations of SKLT and CTP4 followed by morphometric analysis of the opercular bone (Fig. 3A). Highest non-toxic concentrations—31.6 µg/mL for SKLT and 200 µg/mL for CTP4 (Supplementary Table II)—were established based on mortality records. Both extracts induced an increase of the mineralized area of the opercular bone in a dose-dependent manner (Fig. 3B and C), indicating the presence of osteogenic compounds. While CTP4 did not affect the head area (HA) at the concentrations tested, SKLT increased it at 31.6 µg/mL and 56 µg/mL, suggesting that other developmental processes (e.g., cranial development) might be affected (Supplementary Fig. 1). To investigate a possible role of osteoblasts in the pro-osteogenic capacity of microalgae extracts, cellular dynamics were monitored using transgenic reporter lines: *Tg(runx2:EGFP)* for osteo-chondroprogenitor cells, *Tg(sp7:mCherry)* for immature osteoblasts and, *Tg(oc:EGFP)* for mature osteoblasts. A significant increase in the signal associated with *osterix/sp7* (*sp7*^+^ cells) and *osteocalcin* (*oc*^+^ cells), but not of *runt related transcription factor 2* (*runx2*^+^ cells) was observed, suggesting that ethanolic extracts may increase operculum mineralized area through a specific action on committed osteoblast differentiation, but not on early differentiation of mesenchymal precursors (Fig. 4B, D, F). qPCR data confirmed that the expression of *runx2a* and *runx2b* was indeed unaffected in SKLT treated fish, while the expression of *sp7* was increased. Accordingly, expression of *col10a1a* (*collagen type X alpha 1a*), whose expression is directly controlled by *sp7*, was upregulated in SKLT treated fish. In contrast, expression of *sp7*, *oc1* and *oc2* was not affected in CTP4-treated fish (Fig. 4G).

### Dietary exposure to ethanolic extracts promotes bone formation and mineralization, and decrease the incidence of skeletal anomalies

To gain further insights into the osteogenic and osteoblastogenic effects of the extracts, zebrafish were fed diets supplemented with CTP4 and SKLT from 8 to 55 dpf. The five experimental diets produced were homogeneous in terms of proximal composition (Supplementary Table III) and content of P, Ca, K, and Mg (Supplementary Fig. 2). Fish fed SKLT 0.5% and CTP4 2.5% were larger than control fish at 20 dpf (Supplementary Fig. 3), but all fish had similar length and weight at 55 dpf. Our data suggest that none of the extracts significantly affected fish growth.

**Fig. 5:**
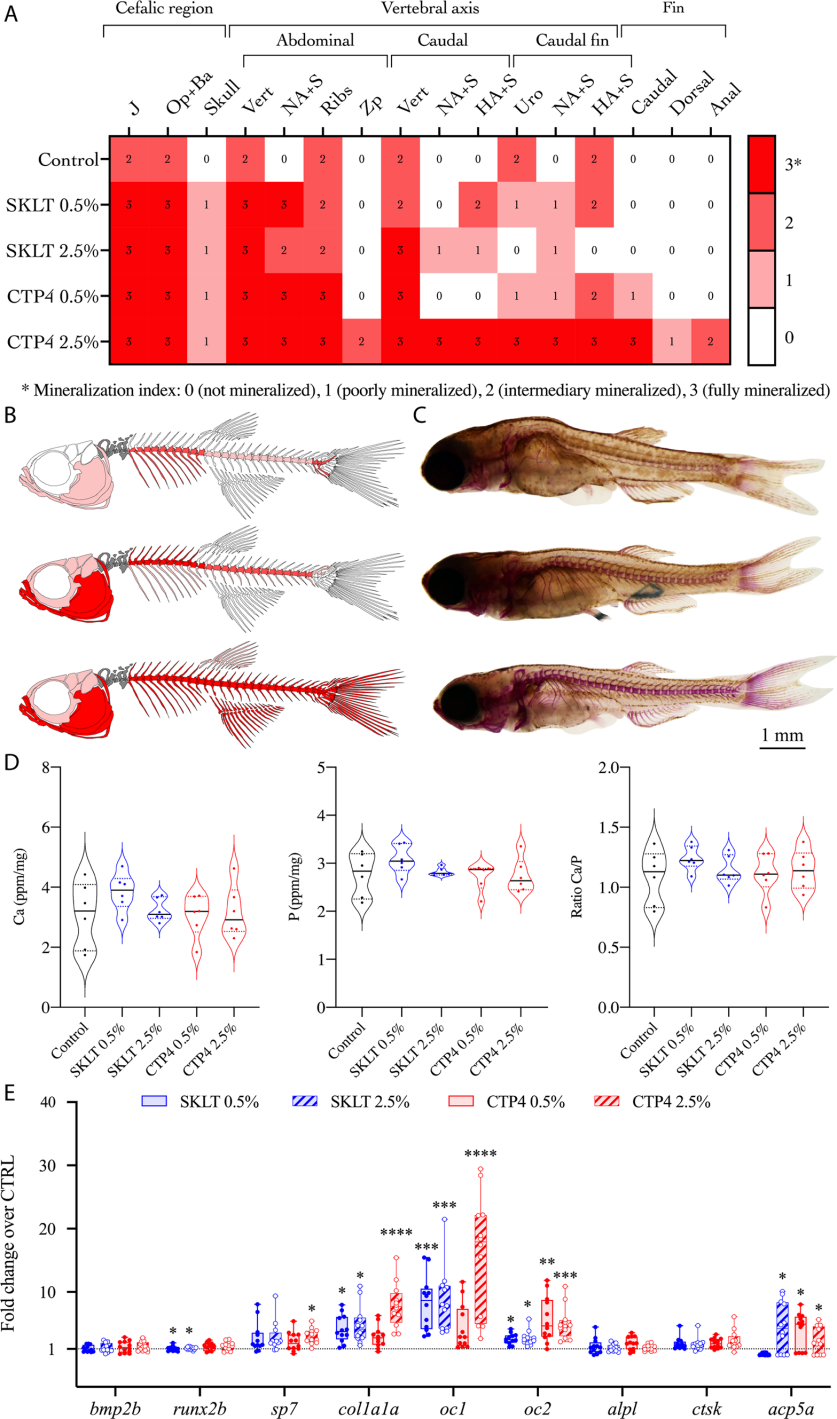
Mineralization status of zebrafish juveniles fed diets supplemented with the ethanolic extracts of *Skeletonema costatum* (SKLT) and *Tetraselmis striata* CTP4 (CTP4). **A** Heatmap displaying the group modal values for the mineralization index assigned to each skeletal structure. **B** Schematic representation of the modal mineralization status of the fish fed CTRL, SKLT 2.5% or CTP4 2.5% (from top to bottom). **C** Representative images of fish illustrated in C. **D** Content of phosphorus (P) and calcium (Ca), and Ca/P ratio in juveniles fed experimental diets. **E** Expression of marker genes for osteoblastic differentiation, ECM mineralization, and osteoclast function. For gene expression, differences were tested through Student’s *t* test. Asterisks indicate *p* < 0.0332 (*), *p* < 0.0021. (**),* p* < 0.0002 (***) and *p* < 0.0001 (****). *J* jaws, *Op + Ba* operculum and branchial arches, *Vert* vertebral bodies, *NA + S* neural arches and spines, *HA + S* haemal arches and spines, *Uro* caudal vertebrae bodies and urostyle

**Fig. 6:**
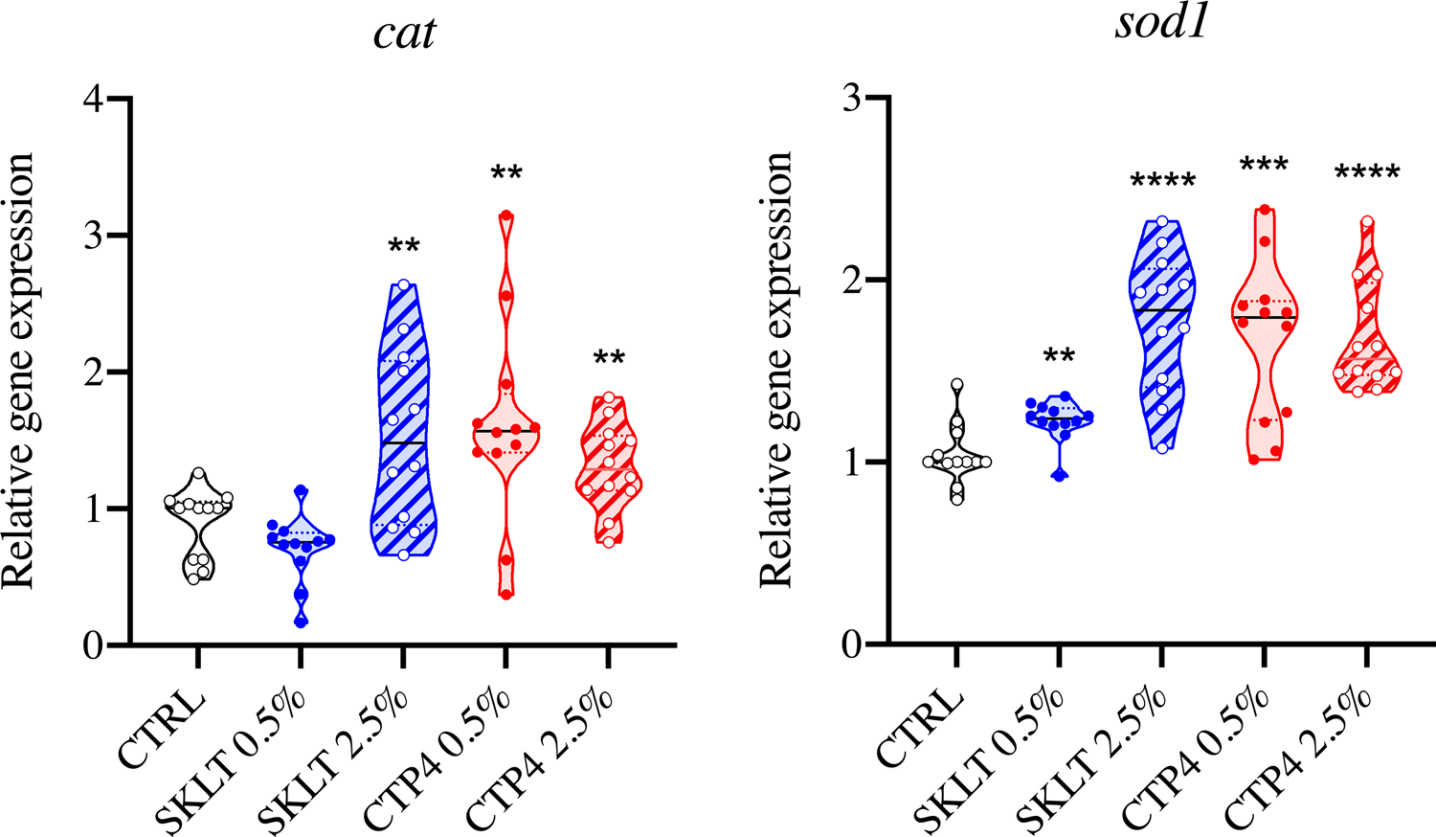
Effect of the ethanolic extracts of *Skeletonema costatum* (SKLT) and *Tetraselmis striata* CTP4 (CTP4) on the expression of antioxidant response markers in juvenile zebrafish (55 dpf). *cat* catalase, *sod1* superoxide dismutase 1, soluble. Statistical differences between each group and the control (CTRL) were tested through Student’s *t* test (*p* < 0.05). Asterisks indicate values statistically different. *p* < 0.002 (**), *p* < 0.0002 (***), *p* < 0.0001 (****)

Although Ca and P contents did not significantly change in fish fed experimental diets (Fig. 5D), a general increase of the mineralization index attributed to each skeletal structure was observed in those fish. In fact, levels of mineralization increased in all the skeletal regions and structures considered and, in a dose-dependent manner for both extracts, with the fish fed SKLT 2.5% and CTP4 2.5% displaying the highest mineralization indexes (Fig. 5A–C). Accordingly, the expression of marker genes for osteoblast differentiation (*sp7*, *oc1*, *oc2*, *col1a1a*) was increased at 55 dpf in a dose-dependent manner and in fish fed the experimental diets (Fig. 5E). In opposition to the data collected after the short exposure, *runx2b* expression was downregulated in fish fed at both supplementation levels in SKLT diets. Interestingly, fish fed SKLT 2.5%, CTP4 0.5% and CTP4 2.5% also displayed an increased expression of *acp5a*, a marker gene for active osteoclasts and bone resorption (Fig. 5E), suggesting that extracts may also affect bone remodeling. The expression of marker genes for antioxidant function (*cat* and *sod1*) was also increased, suggesting a potentiation of the enzymatic removal of Reactive Oxygen Species (ROS) by the extracts (Fig. 6).

**Fig. 7:**
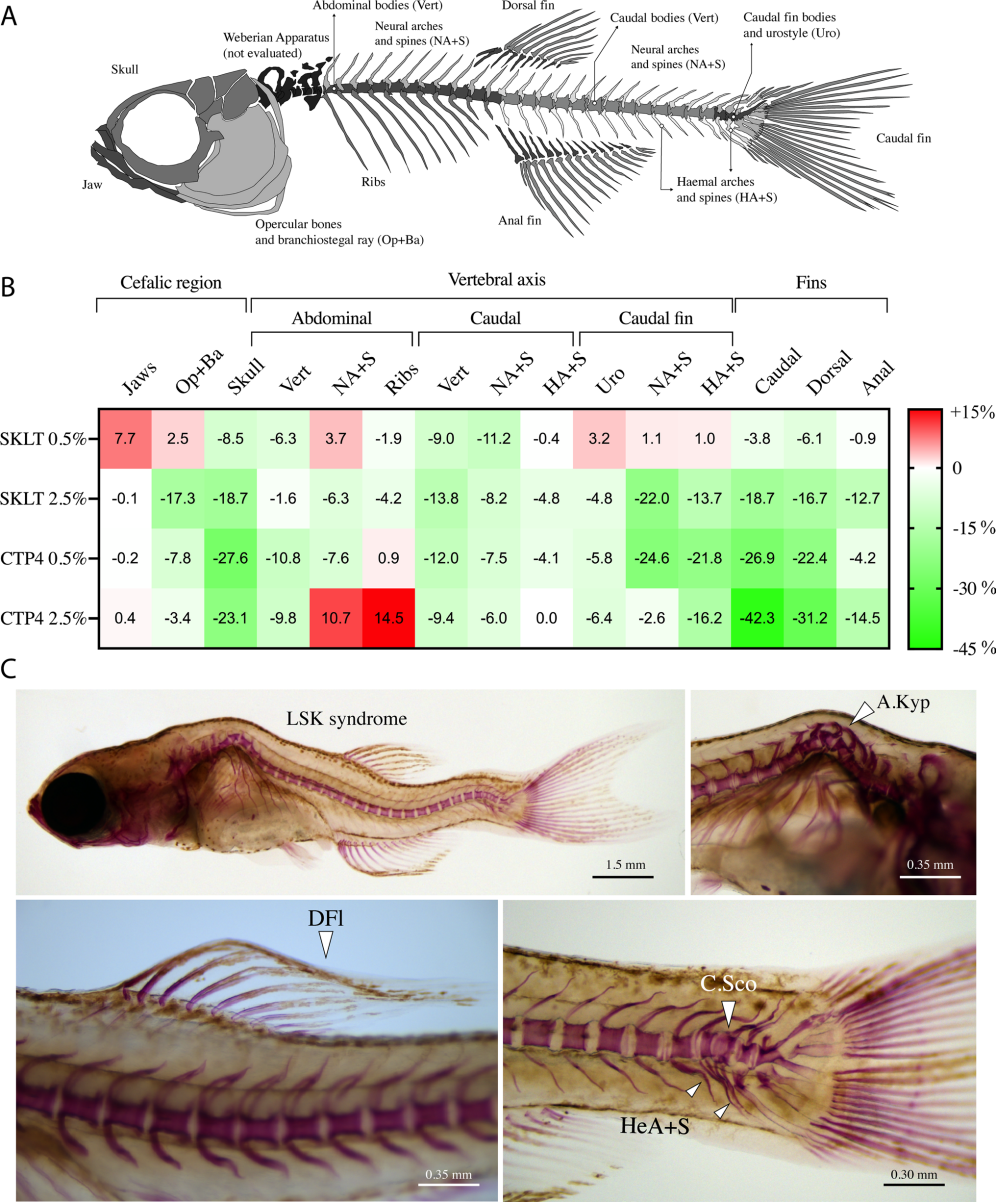
Incidence of skeletal anomalies in zebrafish juveniles fed diets supplemented with the ethanolic extracts of *Skeletonema costatum* (SKLT) and *Tetraselmis striata* CTP4 (CTP4). **A** Scheme illustrating the skeletal elements considered in the analysis of skeletal anomalies. **B** Incidence of anomalies expressed as a percentage of increment/decrement of anomalies relative to the control group (non-supplemented diet). **C** Representative images of commonly found skeletal anomalies in AR-S stained fish for all experimental groups. *Op + Ba* operculum and branchial arches, *Vert* vertebral bodies, *NA + S* neural arches and spines, *HA + S* haemal arches and spines, *Uro* caudal vertebrae bodies and urostyle, *LSK* lordosis–scoliosis–kyphosis, *DFI* deformed fin lepidotrichia, *A.Kyp* abdominal kyphosis, *C.Sco* caudal scoliosis, *HeA + S* deformed haemal arches and spines

The occurrence of skeletal anomalies was assessed in AR-S stained fish and all groups displayed a similar distribution pattern, with opercular bones, abdominal vertebrae, caudal fin complex, and unpaired fins being the most affected structures (Supplementary Fig. 4). A more detailed analysis of the differences in incidence between the levels affecting the individual skeletal structures (Fig. 7A) showed that fish fed experimental diets, in particular SKLT 2.5% and CTP4 0.5%, had a lower incidence of anomalies compared to the control group (Fig. 7B). Interestingly, fish fed CTP4 2.5% had a lower incidence of caudal vertebrae anomalies and anomalies on all the five fins, but a higher incidence of anomalies affecting the skeletal elements associated with abdominal vertebrae (Fig. 7C).

## Discussion

Microalgae species belonging to the genera *Skeletonema* spp. and *Tetraselmis* spp. are commonly cultured worldwide as a food source due to favorable nutritional properties, e.g., high protein contents and diversified fatty acid profiles [39, 40]. Here, we have shown that ethanolic extracts from *Skeletonema costatum* and *Tetraselmis striata* (CTP4 strain) contain compounds with strong pro-osteoblastogenic and pro-mineralogenic activities using in vitro and in vivo fish models. Both extracts demonstrated pro-mineralogenic properties, an effect related to an increased rate of osteoblastic differentiation. This hypothesis is supported by in vivo data collected in zebrafish transgenic lines showing that both extracts selectively increased the populations of both immature (*sp7*^+^) and mature (*oc*^+^) osteoblasts, but not of osteoprogenitor cells (*runx2*^+^). This was further supported by expression data showing a reduced expression of *runx2b* in fish fed SKLT diets. Previous studies have shown that Runx2 regulates osteoblast differentiation in a dualistic manner. It induces the osteoblastic commitment of mesenchymal stem cells at early stages of differentiation but must be downregulated at the final stages of osteoblast maturation because of its capacity to maintain osteoblasts in an immature state [41–44]. We propose that the increase of *sp7* and *oc* expression in larvae exposed to microalgae extracts and the decrease of *runx2b* expression observed in juveniles fed the same extracts is indicative of an accelerated osteoblast maturation that could explain the improved mineralogenic performances observed in exposed fish.

Furthermore, both the larvae exposed to the extracts for a short period (3 days) and the juveniles fed the extracts for a long period (50 days) showed a clear increase in the mineralization of skeletal elements (operculum for larvae and overall skeleton for juveniles). This suggests that the stimulation of osteoblastic differentiation by the microalgae extracts may be then translated into an increased mineralization status. We have also observed that both CTP4 and SKLT diets elevated the expression of genes involved in ECM formation (*col10a1a*, *col1a1a*) and resorption (*acp5a*), suggesting that compounds present in both extracts may have the ability to modulate bone remodeling. This process is highly regulated and governed by molecular programs that mitigate bone anabolic processes by the stimulation of osteoclastic differentiation, which is controlled by both osteoblasts and osteocytes through the RANK-RANKL-OPG signaling pathway [45]. The stimulation of resorptive processes is coherent with an overall increase of bone formation and osteoblastic maturation induced by the extracts. For example, human patients receiving treatment with PTH analogues display an increased osteoclastic differentiation, which is a secondary effect of the activation of anabolic mechanisms for a prolonged time [46].

Fish fed microalgae extracts were also characterized by a reduced incidence of skeletal anomalies in most skeletal elements, indicating an overall improvement of the skeleton health status. Still, an increase in skeletal anomalies associated to the accessory elements of abdominal vertebrae (ribs, neural arches and spines) was observed in fish fed CTP4 2.5%. It might not be a coincidence that this group was also the one presenting the stronger induction of mineralization. In this aspect, it has been shown in zebrafish that conditions that overstimulate bone mineralization, as it is the case for high levels of dietary phosphorus, may trigger an increased incidence of skeletal abnormalities [47].

In this work, phosphorus content was similar in all the experimental diets and cannot be responsible for the increased incidence of skeletal anomalies. Still, an excessive or excessively rapid ossification of the skeletal elements in fish fed CTP4 2.5% may be at the origin of an increased incidence of anomalies in these elements. Fish fed the CTP4 2.5% diet presented the highest degree of mineralization among all groups, and the accessory elements of the abdominal vertebrae showed the highest mineralization index reported. As such, a lower supplementation, for example at 0.5% as tested in this work, may represent a healthier option to provide a better balance between skeletal morphogenic processes and bone mineralization.

Interestingly, the expression of two marker genes of the antioxidant system—the catalase (*cat*) and superoxide dismutase (*sod1*)—was upregulated in fish exposed to both extracts (Fig. 6). It is important to mention that *Skeletonema costatum* and *Tetraselmis striata* CTP4 recently sparked the interest of the scientific community because of their high content in compounds with antioxidant activity, such as polyphenols [39, 48–53]. Ethanolic fractions of *S. costatum* and *T. striata* CTP4—rich in polyunsaturated fatty acids, alkanes and alkenes, long-chain alcohols, esters, ethers, and sterols—were also found to have in vitro radical scavenging activities [50, 52]. We hypothesize that the pro-osteogenic effects observed in fish exposed to microalgae extracts could be explained, at least in part, by the presence of antioxidant compounds in these extracts. Reactive oxygen species (ROS) are known to negatively affect bone mineral status and affect bone cells through several mechanisms, by inducing osteoclastic differentiation while suppressing osteoblastic differentiation and survival, being at the basis of various bone erosive pathologies including age-related osteoporosis [54, 55]. In this regard, the exposure of mammalian and fish models (in vivo and in vitro) to pro-oxidant agents have been shown to inhibit osteoblastic differentiation and activity and increased the incidence of skeletal anomalies, but the supplementation of antioxidants could counteract these negative outputs [56–61]. Recent studies reporting the osteoactive and antioxidative potential of hydroethanolic, methanolic, and dichloromethane extracts prepared from green (*Cladophora rupestris* and *Codium fragile*) and red (*Ceramium secundatum*, *Ceramium pallidum*, and *Plocamium lyngbyanum*) macroalgae, further support this hypothesis [5, 6, 62]. Similarly, polyphenol-rich extracts prepared from marine halophyte plants also triggered pro-osteogenic and pro-mineralogenic activities [7]. The increased expression of *cat* and *sod1* in the present study may indicate a potentiation of fish antioxidant defenses upon exposure to microalgal extracts. In fact, many antioxidant agents exert their protective role against oxidative damage by upregulating the expression of first-line antioxidant enzymes [63–65]. However, this hypothesis should be further confirmed in future studies (i) by gathering data on protein levels and activity for Cat and Sod1, and on markers of cellular oxidative damage (e.g., DNA damage, lipid peroxidation, and loss in liposome membrane stability), but also (ii) by measuring the production of ROS in fish fed SKLT and CTP4 diets and challenged with pro-oxidant compounds.

Non-antioxidant compounds found in high amounts in microalgal biomass and known to stimulate bone morphogenesis and mineralization [66], may also be responsible for the pro-osteogenic effect observed here, e.g., polyunsaturated fatty acids [67, 68], phospholipids [69], liposoluble vitamins such as vitamin D, A and K [70–72], and minerals such as calcium, phosphorus, and magnesium [66, 72]. To identify the osteoactive compounds, extracts should be further fractionated and bioactive fractions should be characterized, e.g., through liquid chromatography coupled to mass spectrometry. Once identified and purified, these compounds should be further validated for applications in aquaculture nutrition as dietary supplements to ameliorate the skeletal health of reared fish species, or in the treatment of human bone erosive pathologies. Thus, future studies should aim at better characterizing the effect of these extracts in animal models that closely recapitulate the phenotypes of human pathologies. In this regard, zebrafish and medaka (*Oryzias latipes*) models of osteoporosis [24–26], osteomalacia [47], and Paget’s disease [73, 74] are available. Ovariectomized rats and mice are the most commonly used models of post-menopausal osteoporosis [75, 76], and rodent models of Paget’s disease are also available [77]. In addition, mammalian models resembling human diseases with secondary bone symptoms, such as vitamin D deficiency [78], hyperparathyroidism [79], and chronic kidney diseases [80] have been developed.

Overall, our data provide strong evidence for the presence of osteoactive compounds in ethanolic extracts of both microalgae, with the ability to stimulate bone mineralization by increasing osteoblast differentiation. The two species of marine microalgae evaluated, *Skeletonema costatum* and *Tetraselmis striata* CTP4, possess a high biotechnological potential as dietary supplement to ameliorate skeletal health in fish reared in aquaculture. In addition, these extracts might serve as a substrate for further identification of novel pharmaceuticals with applications in human medicine, although this will require a careful evaluation of the translatability of these results to mammalian systems that better model human bone disorders.

### Supplementary Information

Below is the link to the electronic supplementary material.

Supplementary file1 (DOCX 3051 KB)

## Data Availability

All datasets generated during and/or analyzed during the current study are available from the corresponding author on reasonable request.
