# Supplementary material for: The osteogenic and mineralogenic potential of the microalgae Skeletonema costatum and Tetraselmis striata CTP4 in fish models
Source: Cell Mol Life Sci. 2023 Sep 30;80(10):310. doi: 10.1007/s00018-023-04953-y (PMC10543572; doi:10.1007/s00018-023-04953-y)
Supplement: Supplementary file 1 — Supplementary file1 (DOCX 3051 KB) [file 18_2023_4953_MOESM1_ESM.docx]

**Supporting Information**

**Supplementary Table I.** qPCR primers used for gene expression in zebrafish larvae and juveniles.

| Gene acronym |  | Primer sequence (5^′^ → 3^′^) | NCBI reference sequence |
| --- | --- | --- | --- |
| *eef1a1l1* | Fw | TTGAGAAGAAAATCGGTGGTGCTG | NM_131263 |
|  | Rev | GGAACGGTGTGATTGAGGGAAATTC |  |
| *actb1* | Fw | GATGCGGAAACTGGCAAAGG | NM_131031 |
|  | Rev | GAGGAGGGCAAAGTGGTAAACG |  |
| *rps18* | Fw | AACACGAACATTGATGGAAGACG | NM_173234 |
|  | Rev | ATTAGCAAGGACCTGGCTGTATTT |  |
| *runx2a* | Fw | AGCCGACCCAGCCCAGTTTGAG | NM_212858 |
|  | Rev | TGGGGTGTAGGTGAATGTTGCTGGATA |  |
| *runx2b* | Fw | TCAGGAATGCCTCAGGGGTTATG | NM_212862 |
|  | Rev | CTTGCGGTGGGTTTGTGAATACT |  |
| *oc1* | Fw | CACTCCTGCTCCTCATGTGC | NM_001083857 |
|  | Rev | GTGTAAGCCGCTACGATCCC |  |
| *oc2* | Fw | TGCTGCCTGATGACTGTGTG | NM_001291889 |
|  | Rev | GTGCAGTTCCAGCCCTCTTC |  |
| *sp7* | Fw | AGCCTCGCATCTGAAAGCCCAC | NM_212863 |
|  | Rev | GTTTTCTGGTGTTTGCTGAGGTGGTC |  |
| *alpl* | Fw | TTCCTCTGCGGTGTCAAAGCCA | NM_201007 |
|  | Rev | AAGCAGCACTCGGGGTGGCAT |  |
| *col10a1* | Fw | TGGAGCGCCTGGAGTTGGTT | NM_001083827 |
|  | Rev | GGCCCAGATTCCCCATCACGG |  |
| *col1a1a* | Fw | GCTTCATTGCCCAGCCACAGGA | NM_199214 |
|  | Rev | GCAGGGTTCTTCTTGGTGCCGTCT |  |
| *ctsk* | Fw | GGATTCCCTCTGCTGGTGCTG | NM_001017778 |
|  | Rev | GCATGTTCTTCTCCCAAATCGTCC |  |
| *acp5a* | Fw | ATAGAGACCGCTACAGCCCGCA | XM_005165726 |
|  | Rev | TGCCAGCAATGACGTACCAAGG |  |
| *cat* | Fw | TATCAGGGATACGCTTCTGTTTCCG | NM_130912 |
|  | Rev | ACTGAACAGGAAAGACACCTGGTG |  |
| *sod1* | Fw | TCCTTCTCATGAATCACCATGGTCC | NM_131294 |
|  | Rev | GCCAACCGATAGTGTGAGACACG |  |

**Supplementary Table II**. Mortality induced on zebrafish larvae following treatment with the two extracts from 3-6 days post fertilization. Mortality is shown as average of 2 or 3 experimental replications. *, higher non-toxic concentration.

| **Treatment** | **Concentration (μg/mL)** | **Mortality** |
| --- | --- | --- |
| SKLT | 1 μg/mL | 0/15 |
|  | 10 μg/mL | 0/15 |
|  | 31.5 μg/mL | 0/15^*^ |
|  | 56 μg/mL | 2/15 |
| Ethanol | 0.1 % | 0/15 |
| Calcitriol | 10 pg/mL | 1/15 |
| CTP4 | 10 | 0/15 |
|  | 31.6 | 0/15 |
|  | 100 | 0/15 |
|  | 200 | 0/15* |
| Ethanol | 0.1 % | 0/15 |
| Calcitriol | 10 pg/mL | 0/15 |

**Supplementary Table III**. Ingredients and proximal composition of the control diet used as base for the production of experimental diets by vacuum coating.

| **Ingredients** | **Relative amount (%)** |
| --- | --- |
| Fish meal | 10.00 |
| Fish gelatin | 4.00 |
| Soy protein isolate | 6.00 |
| Soy protein concentrate | 10.00 |
| Corn gluten meal | 8.50 |
| Soybean meal | 50.00 |
| Vitamins and minerals premix | 1.00 |
| Monoammonium phosphate | 1.50 |
| Fish oil | 2.00 |
| Palm oil | 7.00 |
| **Composition** | **Relative amount (%)** |
| Crude protein | 50.2 |
| Crude fat | 12.3 |
| Fiber | 2.5 |
| Starch | 4.4 |
| Ash | 7.5 |
| Gross energy (MJ/kg feed) | 20.0 |

**
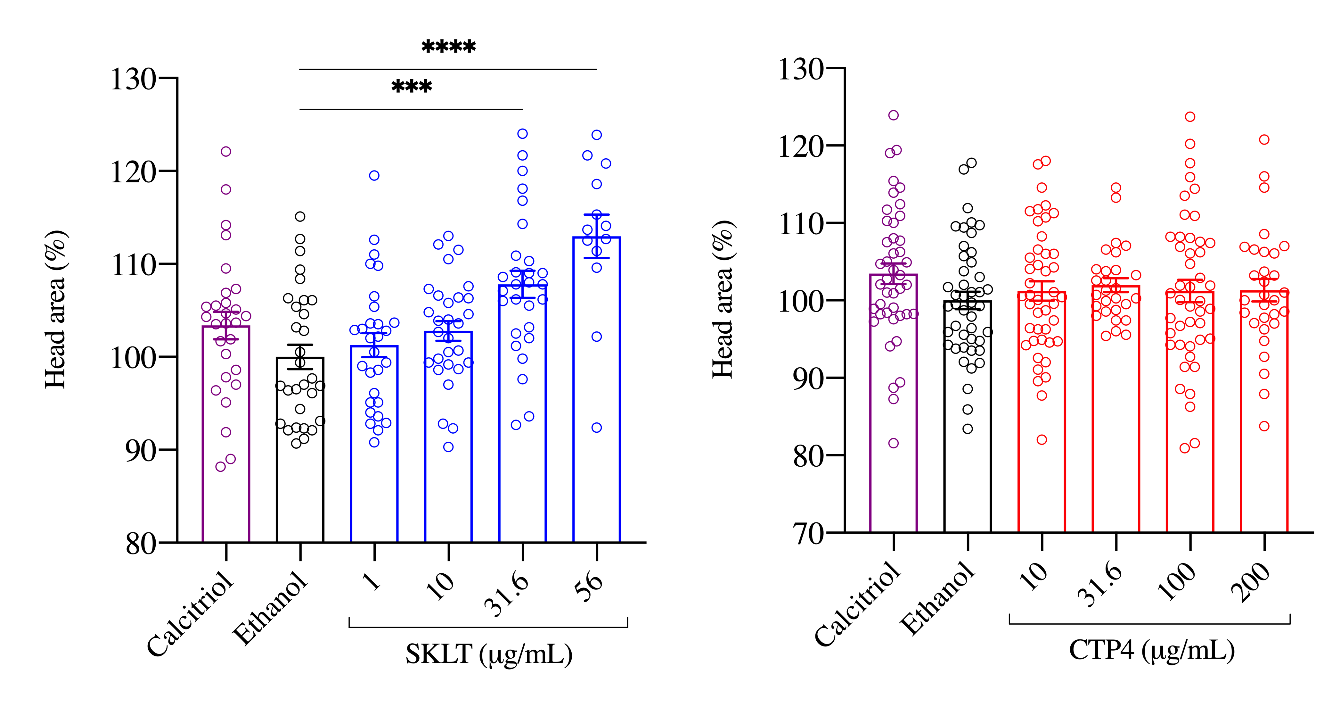
**

**Supplementary Figure 1.** Effect of the ethanolic extracts of *Skeletonema costatum* (SKLT) and *Tetraselmis striata* CTP4 (CTP4) on the area of the head of 6-dpf zebrafish larvae. Values are presented as mean ± SE and are percentages over the control group. Normality was tested through Anderson-Darling test (*p* < 0.05). Statistical differences were tested through one-way ANOVA followed by post-hoc Dunnett’s or Kruskal-Wallis test (*p* < 0.05). Asterisks indicate values statistically different. *p* < 0.0002 (***), *p* < 0.0001 (****).


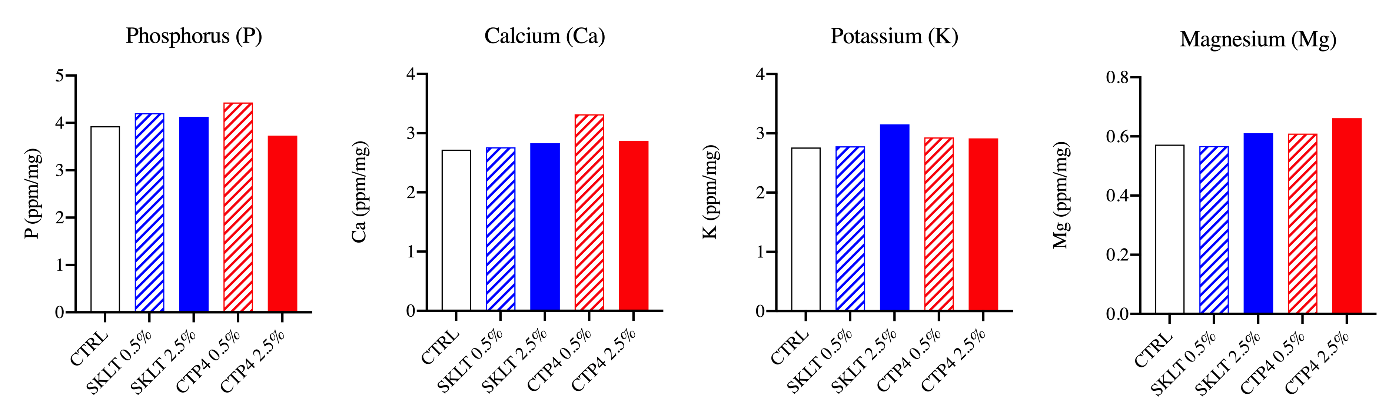


**Supplementary Figure 2.** Content of phosphorus (P), calcium (Ca), potassium (K), and magnesium (Mg) in the five experimental diets used for the feeding trial.

**
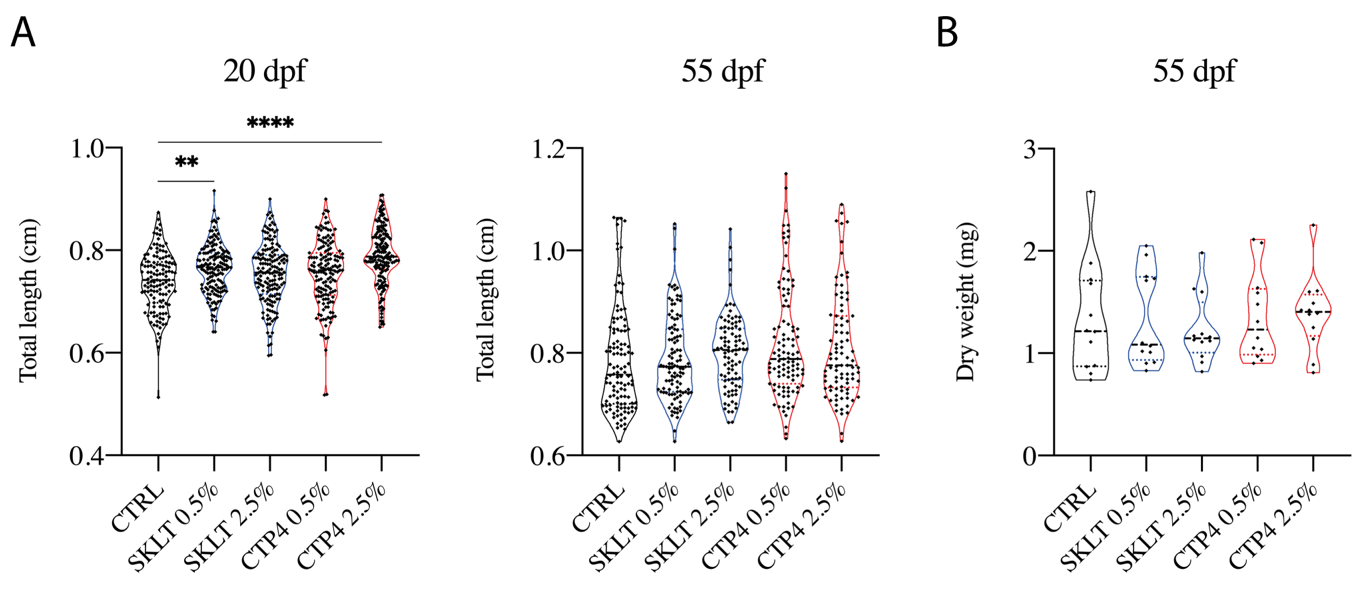
**

**Supplementary Figure 3.** Effect of the ethanolic extracts of *Skeletonema costatum* (SKLT) and *Tetraselmis striata* CTP4 (CTP4) on the total length of 20 and 55 dpf fish (**A**), and the dry weight of 55 dpf fish (**B**). Normality was tested though Anderson-Darling test (*p* < 0.05). Statistical differences were tested through one-way ANOVA followed by post-hoc Dunnett’s or Kruskal-Wallis test (*p* < 0.05). Asterisks indicate values statistically different. *p* < 0.002 (**), *p* < 0.0002 (***).


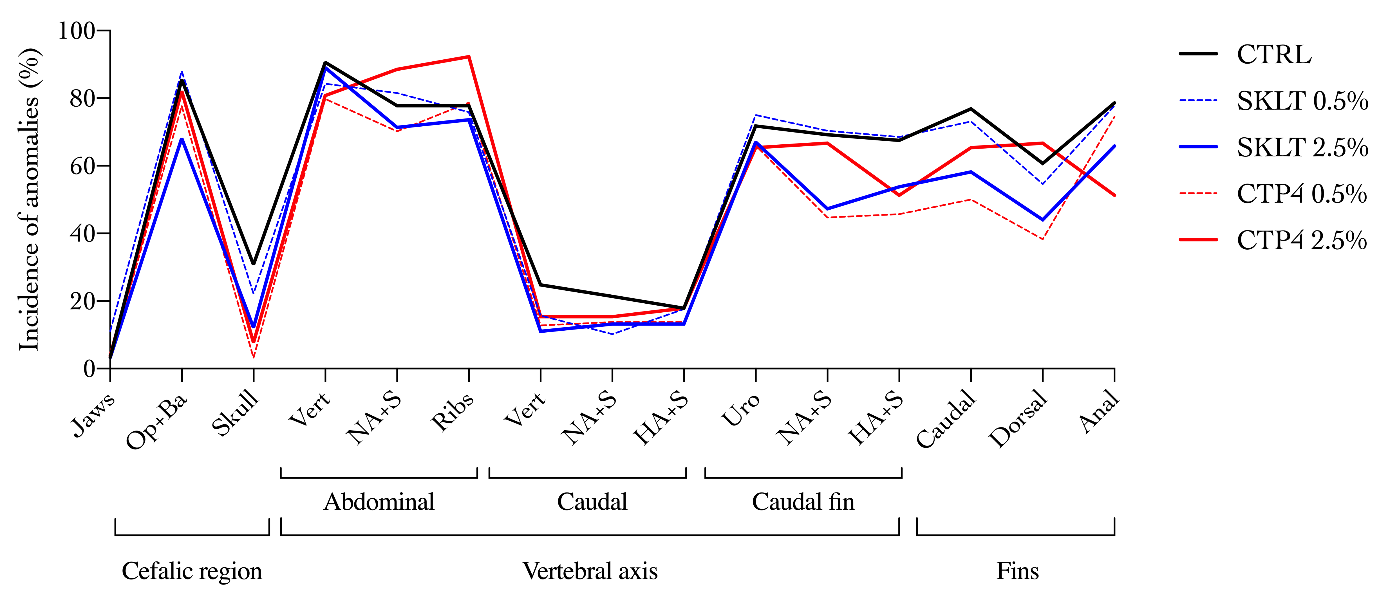


**Supplementary Figure 4.** Effect of the ethanolic extracts of *Skeletonema costatum* (SKLT) and *Tetraselmis striata* CTP4 (CTP4) on the relative distribution of skeletal anomalies in juvenile zebrafish (55 dpf) for each skeletal structure. Op+Ba, operculum and branchial arches; Vert, vertebral bodies; NA+S, neural arches and spines; HA+S, haemal arches and spines; Uro, urostyle and caudal vertebrae bodies.
